# Selective Breeding for Low and High *Varroa destructor* Growth in Honey Bee (*Apis mellifera*) Colonies: Initial Results of Two Generations

**DOI:** 10.3390/insects11120864

**Published:** 2020-12-04

**Authors:** Alvaro De la Mora, Berna Emsen, Nuria Morfin, Daniel Borges, Les Eccles, Paul G. Kelly, Paul H. Goodwin, Ernesto Guzman-Novoa

**Affiliations:** 1 School of Environmental Sciences, University of Guelph, 50 Stone Road East, Guelph, ON N1G 2W1, Canada; delamora@uoguelph.ca (A.D.l.M.); bernaemsen@gmail.com (B.E.); nmorfinr@uoguelph.ca (N.M.); pgkelly@uoguelph.ca (P.G.K.); pgoodwin@uoguelph.ca (P.H.G.); 2 Department of Animal Science, Agricultural Faculty, Ataturk University, Erzurum 25240, Turkey; 3 Ontario Beekeepers’ Association Technology Transfer Program, 185, 5420 Hwy 6 N, Orchard Park Office, Guelph, ON N1H 6J2, Canada; dan.borges@ontariobee.com (D.B.); les.eccles@ontariobee.com (L.E.)

**Keywords:** *Varroa destructor*, *Apis mellifera*, honey bee, deformed wing virus, selective breeding, *Varroa* resistance, colony collapse disorder

## Abstract

**Simple Summary:**

The mite *Varroa destructor* is considered the most damaging parasite of honey bees worldwide. Beekeepers use synthetic chemical products to control mite infestations in colonies, but the parasites soon develop resistance to them, which compromises their control. One alternative control strategy is the development of *Varroa*-resistant honey bees. Therefore, a breeding program was initiated to select for lower and higher rates of *Varroa-*population growth (LVG and HVG, respectively) and deformed wing virus (DWV) levels, which is transmitted by the mites. After two years of bidirectional selection, LVG colonies had *Varroa* population increases over the summer of 1.7 fold compared to 9.6 fold for HVG colonies. Additionally, HVG colonies had higher mite infestation rates in adult bees compared to LVG colonies. DWV presence and levels were higher in HVG colonies than in LVG colonies and winter mortality rates were 26% and 14% for the HVG and LVG bee types, respectively. The results of this study thus far indicate that selection for LVG may result in colonies with lower *Varroa* infestation rates, lower prevalence, and levels of DWV and higher colony winter survivorship. Future work will focus on determining mechanisms responsible for genetic differences and in identifying genes associated with *Varroa*-resistance in honey bees.

**Abstract:**

After two years of bidirectional selection for low and high rates of *Varroa destructor* population growth (LVG and HVG, respectively) in honey bee (*Apis mellifera*) colonies in Ontario, Canada, significant differences between the two genotypes were observed. LVG colonies had *V. destructor* population increases over the summer of 1.7 fold compared to 9.6 fold for HVG colonies by Generation 2. Additionally, HVG colonies had significantly higher mite infestation rates in adult bees compared to LVG colonies for both selected generations. DWV prevalence and levels were significantly higher in HVG colonies than in LVG colonies in Generation 1 but not in Generation 2. Winter mortality rates of Generation 1 colonies were significantly different at 26% and 14% for the HVG and LVG genotypes, respectively. The results of this study thus far indicate that selection for LVG may result in colonies with lower *V. destructor* infestation rates, lower prevalence, and levels of DWV and higher colony winter survivorship. Future work will focus on determining what mechanisms are responsible for the genotypic differences, estimating genetic parameters, and molecular analyses of the genotypes to identify candidate genes associated with resistance to *V. destructor* and DWV that could potentially be used for marker-assisted selection.

## 1. Introduction

The mite *Varroa destructor* is considered the most damaging parasite of the western honey bee (*Apis mellifera*) worldwide. This mite has been frequently associated with the collapse of colonies in numerous countries [1,2,3,4,5]. *V. destructor* feeds upon the hemolymph and fat tissues of honey bees and transmits pathogenic viruses to them [6,7,8]. Additionally, *V. destructor* parasitism compromises immune responses, causes weight loss, shortens lifespan, and decreases honey yields of honey bees [9,10,11,12,13,14].

Another major factor associated with honey bee mortality is deformed wing virus (DWV), which is vectored by *V. destructor* [6,15]. DWV replicates in the mite, reaching high levels that are transmitted to the bees when parasitized by *V. destructor* [16]. DWV infections cause wing deformities and shorten the lifespan of bees [6,17]. Thus, both *V. destructor* and DWV are associated with honey bee mortality and colony losses. In locations with cold and long winters, such as Canada, over-wintering conditions favor the mites and DWV to cause more damage than in countries with less severe winters [18]. 

Most beekeepers use synthetic miticides to control *V. destructor* infestations in colonies, but the mites soon develop resistance to their active compounds, which has compromised their effectiveness [19]. Therefore, there is a need for alternative control strategies. One such strategy could be the development of *Varroa*-resistant strains of honey bees. Several attempts for breeding bees with increased resistance to *V. destructor* have been carried out in Europe and North America [20,21]. In North America, in particular, strains of bees showing some level of resistance to *V. destructor* have been produced in breeding programs to develop a Minnesota hygienic stock selected for increased hygienic behavior [22], a Russian honey bee stock selected for low mite infestation levels [23], a *Varroa*-sensitive hygiene (VSH) stock selected for removal of mite-infested brood [24,25], and an Indiana “mite-biter” honey bee stock selected for grooming behavior [26,27]. 

Developing a winter hardy, mite and virus tolerant stock would greatly contribute to a honey bee Integrated Pest Management (IPM) program, and is probably the most sustainable method of reducing colony losses due to the parasite. A previous study showed a strong correlation between lower *V. destructor* and lower DWV levels in honey bee colonies [28], suggesting that selecting for one trait may also select for the other. Therefore, a breeding program was initiated to select for lower rates of *V. destructor* population growth monitoring infection rates of DWV. This work was implemented in Ontario, Canada, with the collaboration of the Ontario Queen Breeders Association and the University of Guelph’s Honey Bee Research Center. Future work will examine the mechanisms that restrain *V. destructor* and DWV growth and the genes linked to increased resistance against these pathogens. This is a report of the initial results from two generations of bidirectional selection showing divergence in *V. destructor* population growth and DWV levels of a bidirectional selection study after two generations of selection.

## 2. Materials and Methods

### 2.1. Experimental Procedures

Experiments were conducted at apiaries of the Ontario Queen Breeders Association (OQBA) and the University of Guelph, ON, Canada. During the first year in 2018 (Generation 0), more than 300 colonies with queens of different genetic backgrounds equally represented (*A. m. ligustica*, *A. m. carnica*, and Buckfast strain) located in 15 apiaries were evaluated for fallen *V. destructor* mites twice [28]. The colonies were evaluated first in mid-spring (May) and then a second time 16 weeks later in late summer (September). Fallen mites were trapped on sticky papers composed of manila folders coated with Crisco^®^ vegetable shortening placed underneath screened bottom boards of the hives. Averages of three-day fallen mite numbers were done twice in mid-spring and compared with those of late summer. The six colonies with the greatest proportional increase in mite numbers between the two evaluations were designated as high *Varroa* population growth (HVG) colonies, and the six colonies with the smallest proportional increase between the evaluations were designated as low *Varroa* population growth (LVG) colonies. These colonies were preselected to be used for larvae grafting the following year, expecting that at least three of each type would survive the winter. After assessments, all colonies were treated against *V. destructor* infestations using amitraz-impregnated plastic strips (Apivar^®^, Veto-Pharma, Palaiseau, France) as per the manufacturer.

In mid-spring of the following year (May 2019), three HVG and three LVG colonies selected in the previous year were used for larvae grafting to rear approx. 150 queens of each genotype. The six colonies used for larvae grafting were the three with the lowest and highest *V. destructor* population growth, respectively, in the previous season. Two original genetic backgrounds were represented in the queens used for grafting purposes. Two of the three HVG selected colonies were from *A. m. carnica* background and one from the Buckfast strain, whereas for the LVG selected colonies, two were from the Buckfast strain and one from *A. m. carnica* origin. The queens produced were allowed to open mate at a common mating yard isolated at least 5 km from other apiaries. Fifteen colonies each in 10 apiaries of the participating queen breeders were dequeened, and then each was divided into two hives. One HVG queen was introduced into one half of each divided hive and one LVG queen was introduced into the other half. This procedure ensured similar starting mite levels as well as colony and apiary conditions for both genotypes. In total, ca. 150 colonies were developed from queens of each genotype to produce bees of Generation 1. Fallen mite populations were determined with sticky papers as described above after establishing the colonies and then again in late summer. For Generation 1 in 2019, the six most HVG and six most LVG colonies were selected as per Generation 0. These procedures were repeated for a third year (Generation 2 in 2020). Winter mortality was visually assessed for colonies of Generation 1 the following spring after selection and evaluations (March 2020). Winter mortality for colonies of Generation 2 will be evaluated in the spring of 2021. 

### 2.2. Varroa Destructor Infestation Rates in Bees

The selected colonies (the three HVG and three LVG colonies used for grafting) as well as 20 additional randomly chosen colonies of the two genotypes were sampled during late summer to determine mite infestation rates and DWV prevalence and levels. For mite infestation rates, approx. 300 adult bees were collected from each colony in jars containing 70% ethanol and the number of mites per 100 bees was calculated for each sample as per Dietemann et al. [29]. Adults and brood from the same colonies were collected and immediately frozen at −70 °C for future assessment of brood infestation as well as for detection and quantification of DWV. 

### 2.3. DWV Identification and Quantification

Total RNA was extracted from samples of 10 bees per colony using One Step-RNA reagent (Bio Basic, Markham, ON, Canada) following the manufacturer’s instructions. For cDNA synthesis, 2 μg of total RNA was reverse-transcribed using the RevertAid H Minus First Strand cDNA Synthesis Kit (Fermentas Life Sciences, Burlington, ON, Canada), following the instructions of the manufacturer. 

DWV was amplified and quantified in qRT-PCR reactions. The primer sequences and qRT-PCR protocols used to identify DWV type A were those by Di Prisco et al. [30]. PCR reactions were done with a BioRad CFX96™ thermocycler (Bio-Rad Laboratories, Mississauga, ON, Canada) and PowerUp™SYBRgreen™(2X) (Applied Biosystems, Foster City, CA, USA) on 96-well plates (Bio-Rad Laboratories, Mississauga, ON, Canada). Each qPCR reaction of 20 µL contained 2 µL of cDNA, 10 µL of SYBRgreen™, 0.4 µL of reverse and forward DWV primers (200 nM), and 7.2 µL of nuclease free H_2_O. A negative control was included in each run by adding 2 µL of nuclease free H_2_O instead of cDNA, and a positive control from previously identified DWV positive bee sample by qRT-PCR was also included. Each reaction consisted of one cycle at 48 °C for 15 min, one at 95 °C for 10 min, 40 cycles at 95 °C for 15 s and 60 °C for 60 s, followed by one cycle at 68 °C for 7 min. Calibration curves to convert Ct values to DWV gc were done using 300 bp gblocks^®^ (Integrated DNA Technologies, Coralville, IA, USA) that included the sequence of the forward primer, amplicon, and reverse primer. The lyophilized gBlock^®^ was diluted with 50 µL of ds H_2_O to obtain an initial concentration of 10 ng/µL that was used to make serial dilutions from 10^9^ to 10^1^ copies [14]. Using a plot of Ct values versus viral copy number (log_10_), a linear equation was used to calculate the DWV genome copy numbers. Ten-fold serial dilutions using gblocks^®^ were also used to optimize the qRT-PCR reaction to obtain an efficiency of 95–105%. Three technical repetitions were done for each qRT-PCR run. Randomly selected amplicons of presumed DWV were sequenced at the University of Guelph Laboratory services to confirm identity.

### 2.4. Statistical Analyses

The data on fold changes in mite population growth were log-transformed as the data did not comply with normality based on the Shapiro Wilk test. The data on mite infestation rates were square-root transformed and the data on DWV copies were log-transformed for the same reasons. The transformed data were subjected to analyses of variance and Fisher LSD tests to separate means when significance was detected. Selection differentials and responses to selection were calculated. Differences in DWV prevalence were tested with χ^2^ tests. All statistical analyses were performed using the R statistical program [31]. 

## 3. Results

### 3.1. V. destructor Population Growth

**Figure 1:**
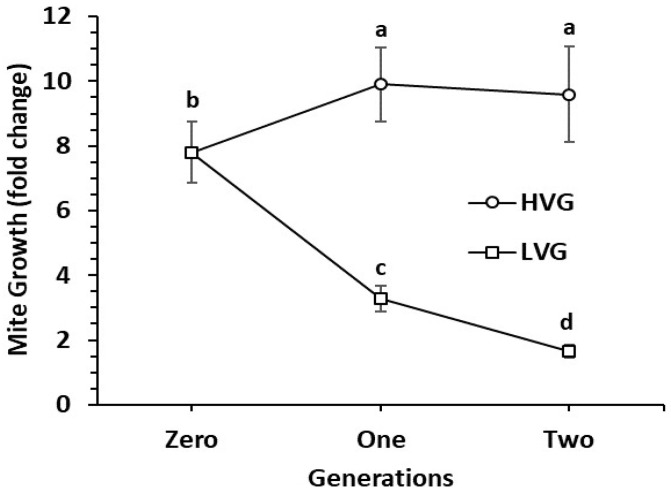
Mean *Varroa destructor* population growth (fold change ± SE) per colony of two honey bee genotypes selected for high and low *Varroa* population growth (HVG and LVG, respectively) during two generations (*n* > 100 per genotype in each generation). Different letters indicate significant differences within and between genotypes.

Mite populations in LVG colonies that are presumably more resistant to *V. destructor* increased 1.7 fold by Generation 2 of selection, which was nearly six times lower than the 9.6-fold mite increase in HVG colonies that are presumed to be more susceptible to *V. destructor* (Figure 1). Significant differences were found for genotype (F_1,646_ = 59.3, *p* < 0.0001), generation (F_2,646_ = 3.2, *p* < 0.05), and genotype × generation interaction (F_2,646_ = 18.9, *p* < 0.0001). Selection differentials (SD) for the colonies used for larvae grafting were 45.5 ± 13.1 and 21.5 ± 11.5 for Generations 1 and 2, respectively, whereas the responses to selection were 6.6 ± 0.8 and 8.0 ± 1.3 for Generations 1 and 2, respectively. Additionally, HVG colonies had significantly higher mite infestation rates in adult bees compared to LVG colonies in the two selected generations (Table 1). There were significant effects of genotype (F_1,76_ = 65.2, *p* < 0.0001), generation (F_1,76_ = 6.7, *p* < 0.05), and genotype × generation interaction (F_1,76_ = 5.7, *p* < 0.05) for this trait. 

### 3.2. DWV Prevalence and Levels

DWV prevalence was significantly higher in HVG colonies than in LVG colonies in Generation 1 (95% versus 65%, respectively, χ^2^ = 5.6, *p* < 0.05) but not in Generation 2 (55% versus 30%, respectively, χ^2^ = 2.6, *p* > 0.05). Similarly, the level of DWV was significantly higher in HVG colonies than in LVG colonies in Generation 1, but not in Generation 2 where there were no significant differences (Table 1). For DWV levels, significant differences were found for genotype (F_1,45_ = 5.7, *p* < 0.05) and generation (F_1,45_ = 34.4, *p* < 0.0001) but not for genotype × generation interaction (F_1,45_ = 2.2, *p* > 0.05). 

### 3.3. Winter Colony Mortality

Winter mortality for Generation 1 colonies was 26% and 14% for the HVG and LVG genotypes, respectively, which differed significantly (χ^2^ = 4.5, *p* < 0.05).

## 4. Discussion

The initial results of this study clearly show that selecting for LVG resulted in significantly lower *V. destructor* infestation rates in colonies than selecting for HVG after two generations of selection. Furthermore, significantly higher winter colony survivorship rates were observed in colonies of the LVG than the HVG genotype. Additionally, LVG colonies had significantly lower prevalence and levels of DWV after one generation of selection than HVG colonies, but these did not significantly differ in the second generation of selection. It is possible that the DWV levels had been affected not only by the selection procedures on the maternal side, but also by the source of drones that mated with the selected queens. It is known that DWV can be transmitted through drone semen [32], which could affect the results of selection since the paternal contribution was not controlled in this study. 

The above results support the hypothesis that a strategy of selecting for reduced *V. destructor* levels in colonies also may have reduced DWV levels and prevalence (in Generation 1), as well as decreased winter mortality of colonies. Thus, selecting for LVG colonies provides multiple benefits to honey bee colonies. These results agree with those of previous work showing decreased rates of *V. destructor* population growth in colonies selected for increased resistance to *Varroa* compared to a nonselected population of Italian bees [27], or bees selected for increased growth of mite populations [33].

Many variables influence *V. destructor* population growth, including climatic factors and genetic factors of the mites and host bees [34,35,36,37,38,39,40,41]. Additionally, infestation rates and *V. destructor* population growth are affected by reinfestation from drifting bees [42,43]. Therefore, future work should investigate the impact of such factors on the *V. destructor* populations in LVG and HVG colonies. 

If the bee genotype is an important contributor to the genetic variability in mite population growth in the colonies, then it should be possible to achieve higher resistance to *Varroa* in selected populations of honey bees by breeding for LVG. Previous studies have shown that *V. destructor* population growth in colonies is a selectable and heritable trait. For example, Lodesani et al. [33] found an estimate of h^2^ = 0.84 for LVG in adult bees, whereas Harbo and Harris [38] found a h^2^ of 1.24 ± 0.49 for the proportion of mites in brood of honey bee colonies in which mite populations were allowed to grow for 10 weeks. More recently, Maucourt et al. [44] also estimated a h^2^ = 0.44 ± 0.56 for rates of *V. destructor* infestation in Canadian honey bee colonies. Future work will be done to determine the heritability estimates of the traits measured in this study. Furthermore, various statistical methods will be used to compare LVG and HVG genotypes when data from additional generations are available. It is possible that the standard errors calculated with ANOVA for the first generation of selection are continued into successive generations, which would then bias the estimation of breeding parameters.

The causes of different rates of infestation with *V. destructor* or infection with DWV between LVG and HVG bees remain to be discovered, and thus work is underway to determine if the divergence between the LVG and HVG genotypes for these traits continues and is sustained over additional generations of selection, and to determine the potential factors and mechanisms involved in causing the divergence in mite population growth between the two selected genotypes. Furthermore, other parameters associated with *V. destructor* resistance can be measured on the two genotypes, such as brood versus adult bee *V. destructor* infestation [38,41,44,45]. Finally, the molecular and cellular basis for lower *V. destructor* populations, DWV prevalence, and DWV levels between LVG and HVG colonies will be later examined, such as by genome sequencing to identify single nucleotide polymorphisms (SNPs) and high-throughput transcript sequencing to identify changes in gene expression related to resistance. This may also allow for the identification of targets for marker-assisted selection for LVG in the future.

## 5. Conclusions

The results of this study thus far are consistent with the idea that selection for LVG can result in colonies having lower *V. destructor* infestation rates, lower DWV prevalence and levels, and higher winter colony survivorship. In 2021, there will be further selection of a third generation of bees for LVG and HVG, as well as analysis of factors that could help explain differences in *V. destructor* population growth in the selected genotypes, as well as estimations of genetic parameters. There will also be a focus on molecular analysis of bees from the third generation of both bee genotypes to pinpoint candidate genes that could be associated with resistance to *V. destructor* and DWV that could be potentially used for marker-assisted selection.

## Figures and Tables

**Table 1:** Percent adult bee infestation with *V. destructor* (± SE) and mean log copies. of deformed wing virus (DWV) (±SE) found in HVG and LVG colonies after two generations of selection. Different letters after means and SE, indicate significant differences between genotypes.

Genotype	Generation	% Infestation	Log Copies of DWV
HVG	One	9.61 ± 1.08 ^a^	8.14 ± 0.42 ^a^
LVG	One	5.15 ± 0.50 ^b^	6.23 ± 0.49 ^b^
HVG	Two	9.15 ± 0.91 ^a^	4.51 ± 0.37 ^c^
LVG	Two	2.62 ± 0.42 ^c^	4.07 ± 0.15 ^c^
